# Supplementary material for: Splice-Junction-Based Mapping of Alternative Isoforms in the Human Proteome
Source: Cell Rep. Author manuscript; Available in PMC 2020 Jan 15. (PMC6961840; doi:10.1016/j.celrep.2019.11.026)
Supplement: 2 [file NIHMS1546469-supplement-2.zip › DF1/PXD006675/LeftAtrium/LeftAtrium_8_SVIL_RPELCTSHSETPTVDDEEK.pdf]

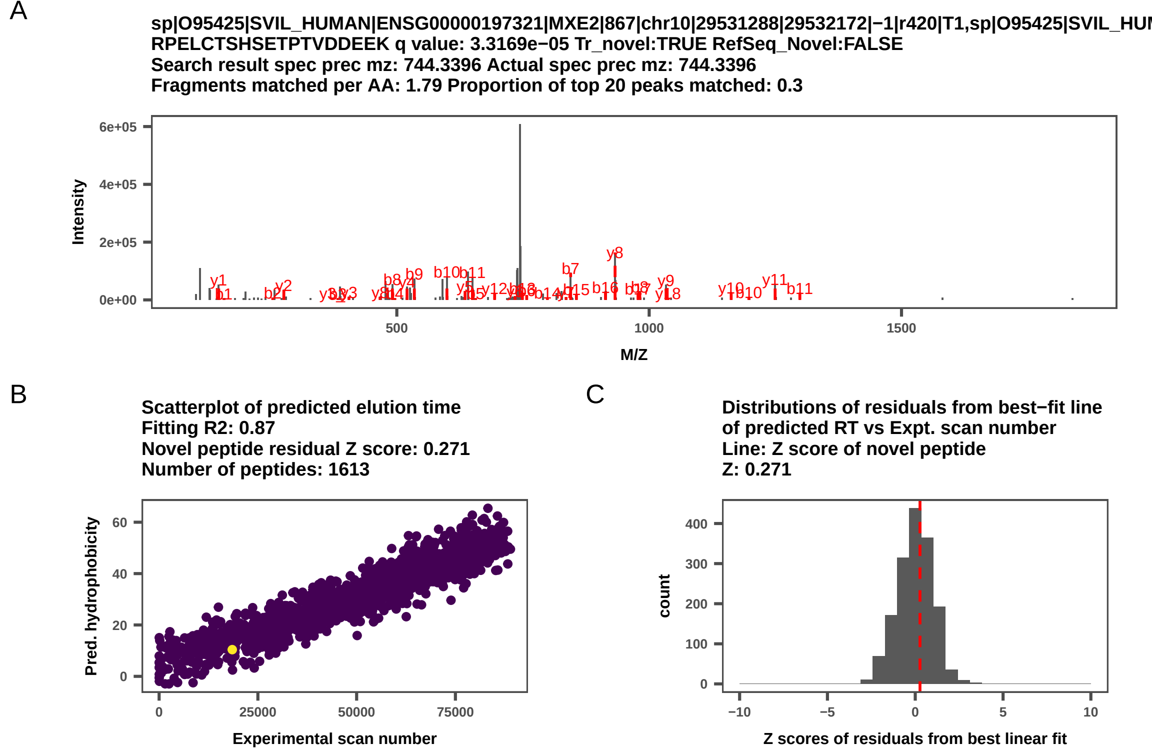
A
sp|O95425|SVIL_HUMAN|ENSG00000197321|MXE2|867|chr10|29531288|29532172|−1|r420|T1,sp|O95425|SVIL_HUM
RPELCTSHSETPTVDDEEK q value: 3.3169e−05 Tr_novel:TRUE RefSeq_Novel:FALSE
Search result spec prec mz: 744.3396 Actual spec prec mz: 744.3396
Fragments matched per AA: 1.79 Proportion of top 20 peaks matched: 0.3
Intensity
6e+05
4e+05
2e+05
0e+00
M/Z
500
1000
1500
y8
b7
b10
b11
b9
y1
y2
y9
y10
y11
b11
b15
b16
b
Scatterplot of predicted elution time
Fitting R2: 0.87
Novel peptide residual Z score: 0.271
Number of peptides: 1613
Pred. hydrophobicity
60
40
20
0
Experimental scan number
0
25000
50000
75000
C
Distributions of residuals from best−fit line
of predicted RT vs Expt. scan number
Line: Z score of novel peptide
Z: 0.271
count
400
300
200
100
0
Z scores of residuals from best linear fit
−10
−5
0
5
10
